# Long-term efficacy and safety of nusinersen in adults with 5q spinal muscular atrophy: a prospective European multinational observational study

**DOI:** 10.1016/j.lanepe.2024.100862

**Published:** 2024-02-06

**Authors:** René Günther, Claudia Diana Wurster, Svenja Brakemeier, Alma Osmanovic, Olivia Schreiber-Katz, Susanne Petri, Zeljko Uzelac, Miriam Hiebeler, Simone Thiele, Maggie C. Walter, Markus Weiler, Tobias Kessler, Maren Freigang, Hanna Sophie Lapp, Isabell Cordts, Paul Lingor, Marcus Deschauer, Andreas Hahn, Kyriakos Martakis, Robert Steinbach, Benjamin Ilse, Annekathrin Rödiger, Julia Bellut, Julia Nentwich, Daniel Zeller, Mohamad Tareq Muhandes, Tobias Baum, Jan Christoph Koch, Bertold Schrank, Sophie Fischer, Andreas Hermann, Christoph Kamm, Steffen Naegel, Alexander Mensch, Markus Weber, Christoph Neuwirth, Helmar C. Lehmann, Gilbert Wunderlich, Christian Stadler, Maike Tomforde, Annette George, Martin Groß, Astrid Pechmann, Janbernd Kirschner, Matthias Türk, Mareike Schimmel, Günther Bernert, Pascal Martin, Christian Rauscher, Gerd Meyer zu Hörste, Petra Baum, Wolfgang Löscher, Marina Flotats-Bastardas, Cornelia Köhler, Kristina Probst-Schendzielorz, Susanne Goldbach, Ulrike Schara-Schmidt, Wolfgang Müller-Felber, Hanns Lochmüller, Otgonzul von Velsen, SMArtCARE Study Group, Christoph Kleinschnitz, Albert C. Ludolph, Tim Hagenacker

**Affiliations:** a Department of Neurology, University Hospital Carl Gustav Carus at Technische Universität Dresden, Dresden, Germany; b Department of Neurology, Ulm University, Ulm, Germany; c Department of Neurology, and Center for Translational Neuro- and Behavioral Sciences (C-TNBS), University Medicine Essen, Essen, Germany; d Department of Neurology, Hannover Medical School, Hannover, Germany; e Friedrich Baur Institute at the Department of Neurology, LMU University Hospital, LMU Munich, Munich, Germany; f Department of Neurology, Heidelberg University Hospital, Heidelberg, Germany; g Department of Neurology, Technical University of Munich, School of Medicine, Munich, Germany; h Department of Child Neurology, Justus-Liebig University Gießen, Gießen, Germany; i Department of Pediatrics, Medical Faculty and University Hospital, University of Cologne, Cologne, Germany; j Department of Neurology, Jena University Hospital, Jena, Germany; k Department of Neurology, University Hospital Würzburg, Würzburg, Germany; l Department of Neurology, University Medicine Göttingen, Göttingen, Germany; m Department of Neurology, Deutsche Klinik für Diagnostik HELIOS Clinic of Wiesbaden, Wiesbaden, Germany; n Translational Neurodegeneration Section “Albrecht Kossel”, Department of Neurology, University Medical Center Rostock, 18147, Rostock, Germany; o Department of Neurology, University of Rostock, Rostock, Germany; p Department of Neurology, University Medicine Halle, Halle (Saale), Germany; q Neuromuscular Diseases Unit/ALS Clinic, Cantonal Hospital St. Gallen, St. Gallen, Switzerland; r Department of Neurology and Center for Rare Diseases, Faculty of Medicine and University Hospital Cologne, University of Cologne, Cologne, Germany; s Department of Neurology, Klinikum Klagenfurt am Wörthersee, Klagenfurt am Wörthersee, Austria; t Department of Neurology, University Hospital Kiel, Kiel, Germany; u Department of Pediatric Neurology, Center for Chronically Sick Children, Charité-Universitätsmedizin Berlin, Berlin, Germany; v Faculty of Medicine and Health Sciences, Carl von Ossietzky University Oldenburg, Oldenburg, Germany; w Department of Neuropediatrics and Muscle Disorders, Medical Center - University of Freiburg, Faculty of Medicine, University of Freiburg, Freiburg, Germany; x Department of Neurology, University Hospital Erlangen, Friedrich-Alexander University Erlangen-Nuremberg (FAU), Germany; y Pediatrics and Adolescent Medicine, Faculty of Medicine University Hospital Augsburg, Augsburg, Germany; z Department of Pediatrics and Pediatric Neurology, Clinic Favoriten, Vienna, Austria; aa Department of Neurology and Epileptology, Hertie Institute for Clinical Brain Research, University Hospitals Tubingen, Tubingen, Germany; ab Department of Pediatrics, Paracelsus Medical University, Salzburg, Austria; ac Department of Neurology, University Hospital Münster, Münster, Germany; ad Department of Neurology, University of Leipzig Medical Centre, Leipzig, Germany; ae Division of Neurology, Medical University Innsbruck, Innsbruck, Austria; af Department of Pediatric Neurology, Saarland University Hosptial, Homburg, Germany; ag Department of Neuropaediatrics, University Children's Hospital, Ruhr-University Bochum, Bochum, Germany; ah Initiative SMA der Deutschen Gesellschaft für Muskelkranke, Freiburg, Germany; ai Department of Paediatric Neurology, Center for Neuromuscular Disorders in Children and Adolescents, Center for Translational Neuro- and Behavioral Sciences, University Hospital, University of Duisburg-Essen, Essen, Germany; aj Department of Neuropediatrics, Dr. v. Haunersche Kinderklinik, University Children's Hospital, Ludwig-Maximilians-Universität München, München, Germany; ak Children's Hospital of Eastern Ontario Research Institute, Division of Neurology, Department of Medicine, The Ottawa Hospital; and Brain and Mind Research Institute, University of Ottawa, Ottawa, Canada; al Institute of Medical Informatics, Biometrics, and Epidemiology, University Hospital Essen, Essen, Germany; am Deutsches Zentrum für Neurodegenerative Erkrankungen (DZNE), Dresden, Dresden, Germany; an Clinical Cooperation Unit Neurooncology, German Cancer Consortium (DKTK), German Cancer Research Center (DKFZ), Heidelberg, Germany; ao Deutsches Zentrum für Neurodegenerative Erkrankungen (DZNE), Rostock/Greifswald, Rostock, Germany; ap Deutsches Zentrum für Neurodegenerative Erkrankungen (DZNE), Ulm, Ulm, Germany; aq Department of Neurological Intensive Care and Rehabilitation, Evangelisches Krankenhaus Oldenburg, Oldenburg, Germany; ar Center for Clinical Trials, University Hospital Essen, Essen, Germany

**Keywords:** Antisense oligonucleotide, Intrathecal therapy, Motor neuron disease, Nusinersen, Spinal muscular atrophy

## Abstract

**Background:**

Evidence for the efficacy of nusinersen in adults with 5q-associated spinal muscular atrophy (SMA) has been demonstrated up to a period of 16 months in relatively large cohorts but whereas patients reach a plateau over time is still to be demonstrated. We investigated the efficacy and safety of nusinersen in adults with SMA over 38 months, the longest time period to date in a large cohort of patients from multiple clinical sites.

**Methods:**

Our prospective, observational study included adult patients with SMA from Germany, Switzerland, and Austria (July 2017 to May 2022). All participants had genetically-confirmed, 5q-associated SMA and were treated with nusinersen according to the label. The total Hammersmith Functional Motor Scale Expanded (HFMSE) and Revised Upper Limb Module (RULM) scores, and 6-min walk test (6 MWT; metres), were recorded at baseline and 14, 26, and 38 months after treatment initiation, and pre and post values were compared. Adverse events were also recorded.

**Findings:**

Overall, 389 patients were screened for eligibility and 237 were included. There were significant increases in all outcome measures compared with baseline, including mean HFMSE scores at 14 months (mean difference 1.72 [95% CI 1.19–2.25]), 26 months (1.20 [95% CI 0.48–1.91]), and 38 months (1.52 [95% CI 0.74–2.30]); mean RULM scores at 14 months (mean difference 0.75 [95% CI 0.43–1.07]), 26 months (mean difference 0.65 [95% CI 0.27–1.03]), and 38 months (mean difference 0.72 [95% CI 0.25–1.18]), and 6 MWT at 14 months (mean difference 30.86 m [95% CI 18.34–43.38]), 26 months (mean difference 29.26 m [95% CI 14.87–43.65]), and 38 months (mean difference 32.20 m [95% CI 10.32–54.09]). No new safety signals were identified.

**Interpretation:**

Our prospective, observational, long-term (38 months) data provides further real-world evidence for the continuous efficacy and safety of nusinersen in a large proportion of adult patients with SMA.

**Funding:**

Financial support for the registry from 10.13039/100005614Biogen, 10.13039/100004336Novartis and 10.13039/100004337Roche.

**Research in context**

**Evidence before this study**

Our study relates to the latest scientific knowledge on “nusinersen in adult patients with 5q-spinal muscular atrophy”. While effectiveness of nusinersen has been demonstrated in children with SMA in randomized double-blind trials before approval, there is still little data regarding the effectiveness in adults. Using PubMed and the search terms “nusinersen”, “SMA” and “adult patients” in the period from March to May 2023, only few real-world evidence studies as well as two reviews and meta-analyses were found. Merely studies that depicted motor function using validated motor scores were taken into account. Most real-world data report positive changes on at least one of the functional measures (such as HFMSE, RULM, 6 MWT) in SMA patients treated with nusinersen. An increase in HFMSE score with a pooled mean change of 1.87 (95% CI 1.05–2.68) was determined through a meta-analysis in treated adult SMA patients, while previous studies showed that untreated adult patients experience deterioration in motor function over time. The results were reported to remain stable over the long term. However, prospective observational studies report efficacy and safety of nusinersen in adults with SMA for a period of no longer than 16 months.

**Added value of this study**

In this prospective, observational, multicentre study in Germany, Austria and Switzerland we provided evidence for the safety and efficacy of nusinersen treatment in adults with SMA for 38 months. All outcome measures (HFMSE, RULM, 6 MWT) showed significant increases compared to baseline throughout the course. Our results demonstrate sustained efficacy of nusinersen on motor function across the phenotypic spectrum of adult SMA, including ambulatory and non-ambulatory patients across a wide age range. Thus, our study shows that the course of the disease in treated SMA patients differs from the natural course.

**Implications of all the available evidence**

Nusinersen in the treatment of adult patients stabilises the progressive disease course of SMA in sitters and may provide continuous benefit in walkers thus remaining a relevant therapeutic option in the long-term course.

## Introduction

5q-associated spinal muscular atrophy (SMA) is an autosomal-recessive inherited neuromuscular disease with an incidence of about 1 per 7000 live births in German speaking countries.^1^ In most cases, a homozygous deletion in the *Survival of Motor Neuron 1* gene *(SMN1)* causes a critical reduction in the functional SMN protein, leading to continuous degeneration of lower motor neurons with progressive muscle denervation over the lifetime.^2^, ^3^, ^4^ The SMA phenotype is characterised by muscle weakness and atrophy of skeletal muscles including deficits of more proximal than distal and trunk muscle groups.^5^ Depending on the severity of the SMA type, the natural disease course includes highly variable phenotypes ranging from newborns who are unable to gain motor milestones and/or respiration without support to patients who are still able to walk at older ages.^6^ The severity is strongly associated with the number of centromeric copies of *SMN1*, the *Survival of Motor Neuron 2* gene (*SMN2*) copies.^7^ A point mutation on position 6 of exon 7 in *SMN2* leads to altered splicing of *SMN2* pre-mRNA.^8^ Additionally, an intronic splicing silencer N1 (ISS-N1) exacerbates exon skipping of exon 7, both resulting in only low functional SMN protein levels derived from the *SMN2* gene.^9^

Nusinersen is an antisense oligonucleotide that increases the expression of *SMN2* by blocking ISS-N1 of *SMN2* pre-mRNA.^10^ Since the approval of this first gene-modifying therapy for SMA in December 2016 by the FDA and in May 2017 by the EMA, the phenotypic landscape of SMA has profoundly changed. However, little efficacy and safety data on adults with SMA were available before approval. In a prospective, observational, multicentre study in Germany over a treatment period of 14 months, we provided evidence for the safety and efficacy of nusinersen treatment in adults with SMA.^11^ Additional studies including meta-analyses provided more evidence.^12^, ^13^, ^14^ However, studies with longer observational intervals are needed to provide more robust conclusions. We have therefore investigated the safety and efficacy of nusinersen in adults with SMA over a longer time period in a larger cohort.

## Methods

### Study design and population

Patients (aged 16–71 years) with SMA participating in the SMArtCARE registry^15^ were included in this prospective, observational, multicentre, study. Recruitment took place between July 2017 and May 2022 within Germany, Switzerland, and Austria. Inclusion criteria were genetically-confirmed, 5q-associated SMA due to homozygous deletion of exons 7, 8, or both, or to compound heterozygous *SMN1* mutations, and nusinersen treatment administered continuously according to the official prescribing information with a minimum treatment time of 14 months. All patients treated with nusinersen and willing to participate in the SMArtCare registry at each centre were included. To avoid selection bias, no other criteria were defined. Study approval was obtained from the local ethics committees of all participating sites (lead Ethics Committee of the University of Freiburg, Germany (EK 56/18)). All patients provided written informed consent.

### Procedures

In all patients, nusinersen (12 mg) was administered intrathecally in accordance with the label, including a loading phase followed by maintenance dosing every 4 months. Intrathecal injections by a trained neurologist or neuroradiologist were given via conventional, fluoroscopy-, ultrasound-, or CT-guided lumbar puncture, based on the individual decision of the treating physician. All patients were treated according to the current standard of care. Adverse events were recorded using a standardised protocol. Motor function assessments were done according to the recommendations of the SMArtCARE registry initiative.^15^ Site evaluators were trained by experienced physiotherapists from the SMArtCARE initiative from Freiburg University (Freiburg, Germany).

### Outcomes and measures

The primary endpoint was the change from baseline in the total Hammersmith Functional Motor Scale Expanded (HFMSE) score at 14, 26, and 38 months. The HFMSE consists of 33 items for motor functions to assess activities of daily living. Each item is scored on a scale from 0 to 2, with higher scores indicating better motor function, up to a maximum of 66 points. A score change of at least three points is considered to be clinically meaningful.^16^ Secondary endpoints were the change from baseline to 14, 26, and 38 months in the Revised Upper Limb Module (RULM) score (20 items with a maximum of 37 points, with higher scores indicating better arm function, and a score change of at least two points considered to be clinically meaningful), and the 6-min walk test (6 MWT; measures the distance (m) a patient is able to walk within 6 min; a change of at least 30 m is considered to be clinically meaningful). Adverse drug reactions were evaluated and reported according to MedDRA (version 25.0).

### Statistical analyses

Statistical analyses were based on pre-post comparisons from baseline to 14, 26, and 38 months, using the estimate of the pre-post differences for primary and secondary endpoints together with the corresponding 95% confidence intervals (CI) and by using the paired sample t-test. In addition to the t-tests, Wilcoxon signed rank test were performed for the pre-post differences in primary and secondary endpoints. As no adjustment was done for the secondary endpoints, the p-values presented are to be interpreted on a descriptive basis only. Correlations were analyzed by Spearman's rank correlation coefficient with α = 0.05. Correlation coefficients r < 0.3 were described as negligible.^17^ Subgroup analyses included SMA type (2 vs. 3) (types 1 and 4 were not separately analysed because of the limited data set), status of ambulation (yes vs. no), and previous spondylodesis (yes vs. no). Subgroup analyses were conducted using the paired sample t-test; for group comparison, the Mann–Whitney U test was used. A mixed model was used to estimate the effect on the HFMSE score. The model was set up with sex, age, spondylodesis, SMA type, ambulatory status, and time as fixed effects, and patient as the random effect. Outliers were not removed because there were no indications of incorrect measurements.

The main results were based on complete case analysis. Because of (the mentioned) missing values we additionally performed an exploratory sensitivity analysis in which the missing data were substituted by 100-fold multiple imputation with the fully conditional specification (FCS) method to stress results of primary analysis. The missing data in the HFMSE differences at 14 months, at 16 months and at 38 months of the primary endpoints were imputed. The imputation process included the following variables: gender, age, spondylodesis, SMA type, HFMSE baseline value and HFMSE scores at the respective time point as well as HFMSE scores at the next and previous time point. After imputation, the patients who discontinued treatment were reset as missing from the imputed data. The analysis for the primary endpoints was carried out repeatedly on the imputed data. As results on the imputed data sets, we obtained combined parameter estimates. Analyses were performed using SAS version 9.4.

### Role of the funding source

Data collection and analysis was carried out by the academic SMArtCARE network, independent of the commercial partner.

## Results

**Fig. 1:**
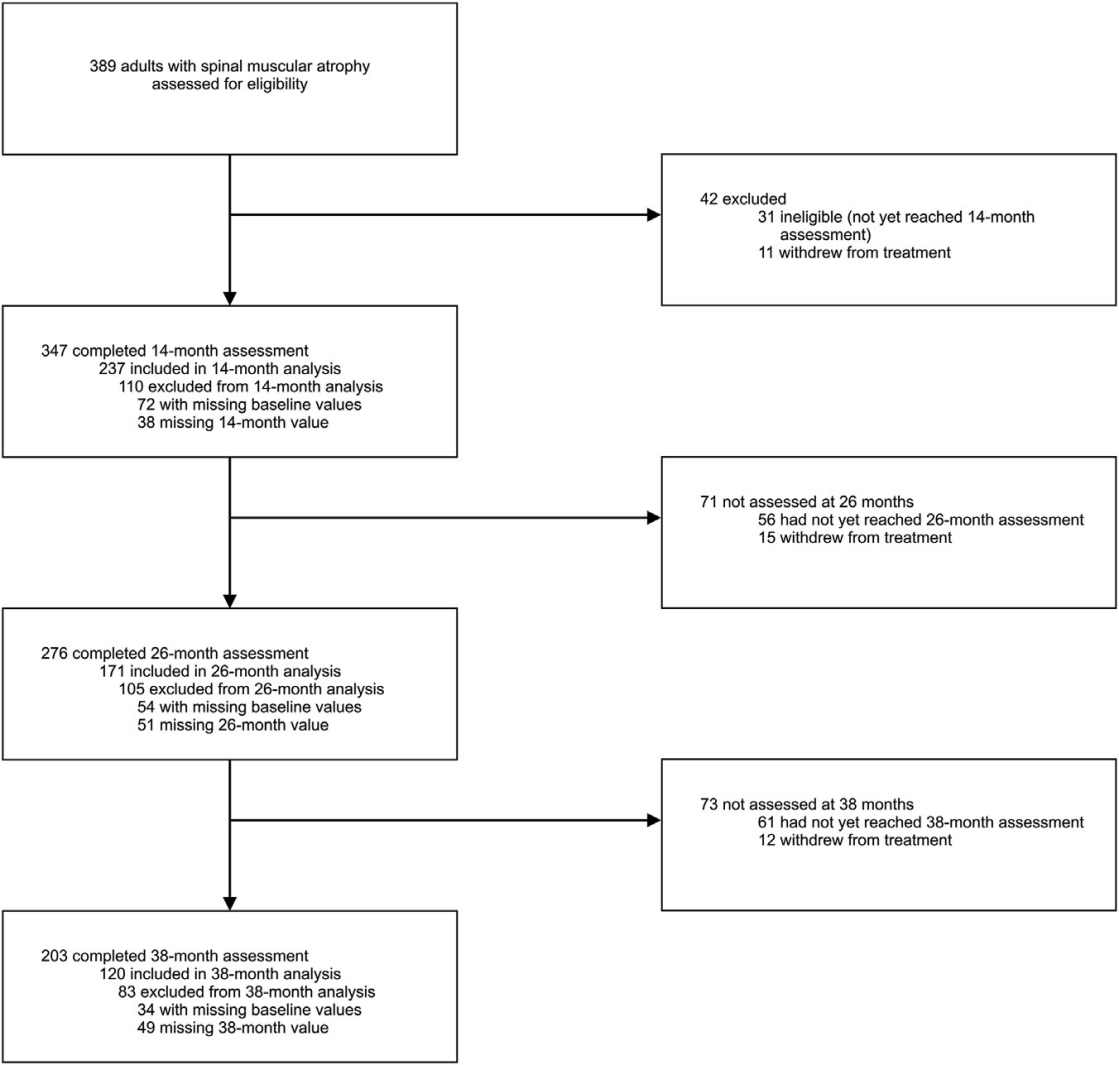
Study profile.

Overall, 389 adult patients with SMA were screened for eligibility for the primary outcome (Fig. 1). 11 patients withdrew from nusinersen treatment before completing the 14-month assessment timepoint. Of whom 4 patients changed treatment to risdiplam. In 5 patients 21 adverse events were documented; one patient suffered from an aseptic meningitis. None of the patients was able to walk; 4 of the 11 patients were type 1.

Thirty-one patients were not included in the analysis because they had not yet reached the 14-month assessment timepoint. In total, 347 completed the 14-month assessment, 276 completed the 26-month assessment, and 203 completed the 38-month assessment (Fig. 1). Data from patients with missing values (e.g., because the patient declined functional testing, there was a competing disease, or no scoring was done either at baseline or 14 months) were excluded from the analysis. The primary endpoint analysis included 237 patients with a treatment period of at least 14 months, 171 patients with a treatment period of 26 months, and 120 patients with a treatment period of 38 months (Fig. 1). The demographic and clinical baseline data of these patients are presented in Table 1.

**Table 1:** Demographic and clinical baseline data of the study cohort.

	14-month analysis	26-month analysis	38-month analysis
No. patients, n (%)	237 (100)	171 (100)	120 (100)
Sex, n (%)			
Female	102 (43)	68 (40)	49 (41)
Male	135 (57)	103 (60)	71 (59)
Age (years), mean ± SD (range)	36 ± 13 (16–71)	35 ± 13 (16–68)	36 ± 13 (16–68)
*SMN2* copy, n (%)			
2	12 (5)	12 (7)	8 (7)
3	82 (35)	55 (32)	36 (30)
4	90 (38)	66 (39)	45 (38)
5	5 (2)	3 (2)	1 (1)
6	3 (1)	2 (1)	1 (1)
7	1 (0)	1 (0)	1 (1)
Unknown	44 (19)	32 (19)	28 (23)
SMA type, n (%)			
1	5 (2)	3 (2)	2 (2)
2	67 (28)	44 (26)	33 (27)
3	156 (66)	117 (68)	83 (69)
4	9 (4)	7 (4)	2 (2)
Ambulant, n (%)	100 (42)	77 (45)	57 (48)
Spondylodesis, n (%)	45 (19)	40 (23)	26 (22)
Baseline Scores, mean ± SD			
HFMSE score	25.3 ± 21.7	26.3 ± 21.7	26.2 ± 21.5
RULM score	24.0 ± 12.5	24.8 ± 12.0	24.9 ± 12.5
6 MWT	333.6 ± 178.9	344.5 ± 183.2	341.0 ± 181.6

Maximum score values: HFMSE_max_ = 66; RULM_max_ = 37.

6 MWT, 6-min walk test; HFMSE, Hammersmith Functional Rating Motor Scale Expanded; RULM, Revised Upper Limb Module; SD, standard deviation.

**Fig. 2:**
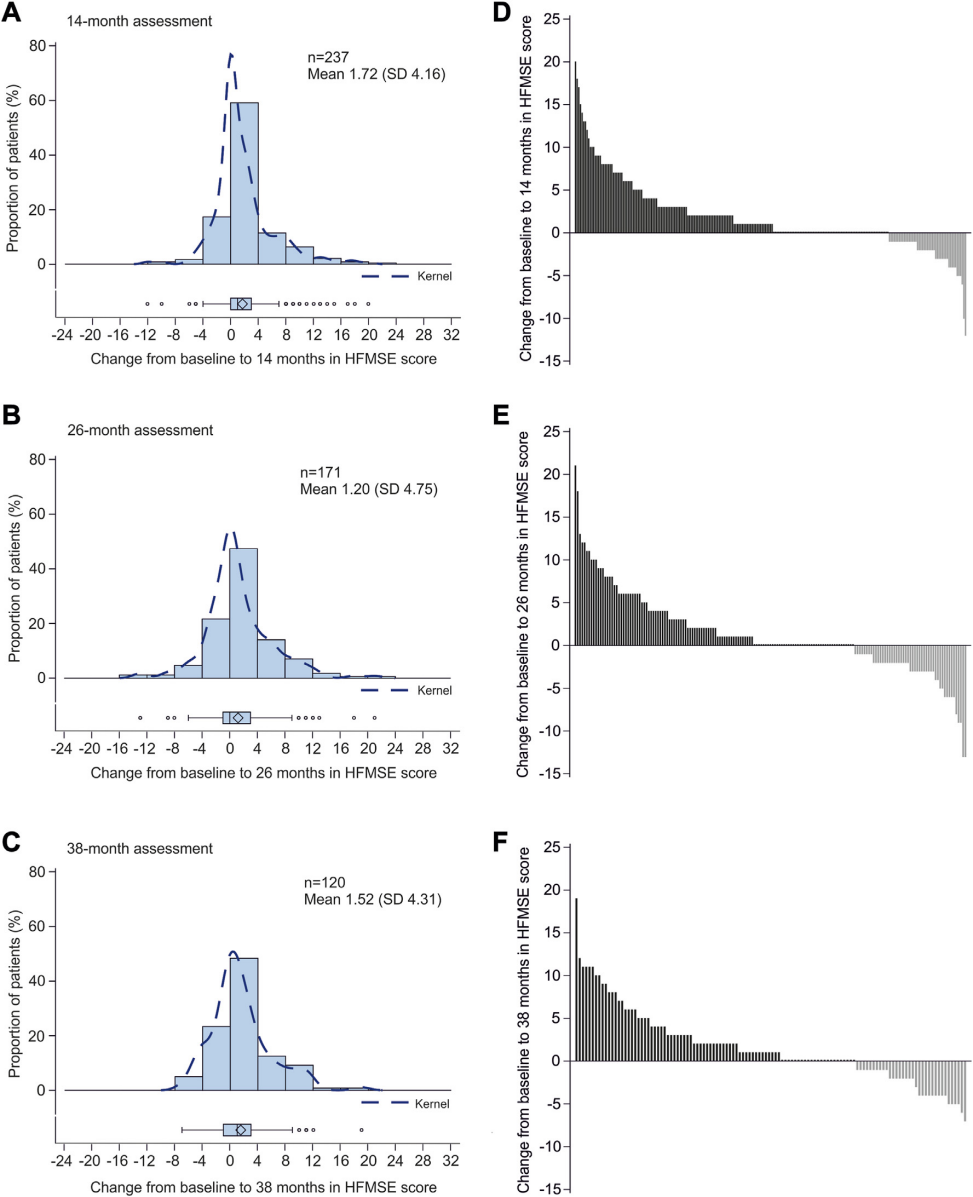
Changes in HFMSE score from baseline to 14 (A, D), 26 (B, E), and 38 (C, F) months. Left panels show distribution of changes in HFMSE score from baseline to 14, 26, and 38 months, with each bar representing the proportion of patients who had improved/worsened. The dashed line is the kernel density estimator for the distribution. Box and whisker plots show median (central line), IQR (boxes), and 1.5 × IQR (whiskers), with individual points representing outliers (those outside of 1.5 × IQR from the median). Diamonds indicate mean values. Panels on the right show changes in HFMSE in individual patients from baseline. Each bar represents a single patient. HFMSE = Hammersmith Functional Motor Scale Expanded; IQR = interquartile range.

**Supplementary Figure S1:**
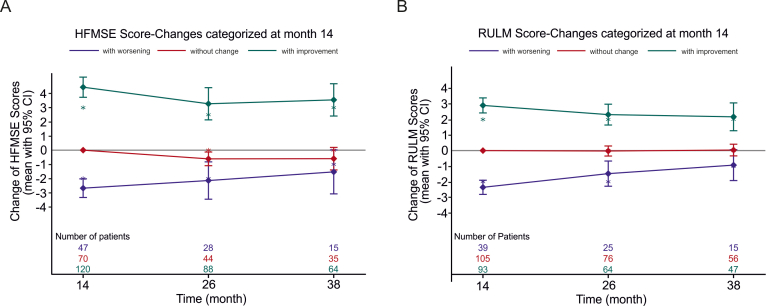
**Supplement Figure 1.** (A) Further course of the subgroups with improvement (>0 points) (green), without change (=0 points) (red), and with worsening (<0 points) (blue) at 14 months compared to baseline in HFMSE score. (B) Further course of the subgroups with improvement (>0 points) (green), without change (=0 points) (red), and with worsening (<0 points) (blue) at 14 months compared to baseline in RULM score. HFMSE = Hammersmith Functional Motor Scale Expanded; RULM = Revised Upper Limb Module.

**Supplementary Figure S2:**
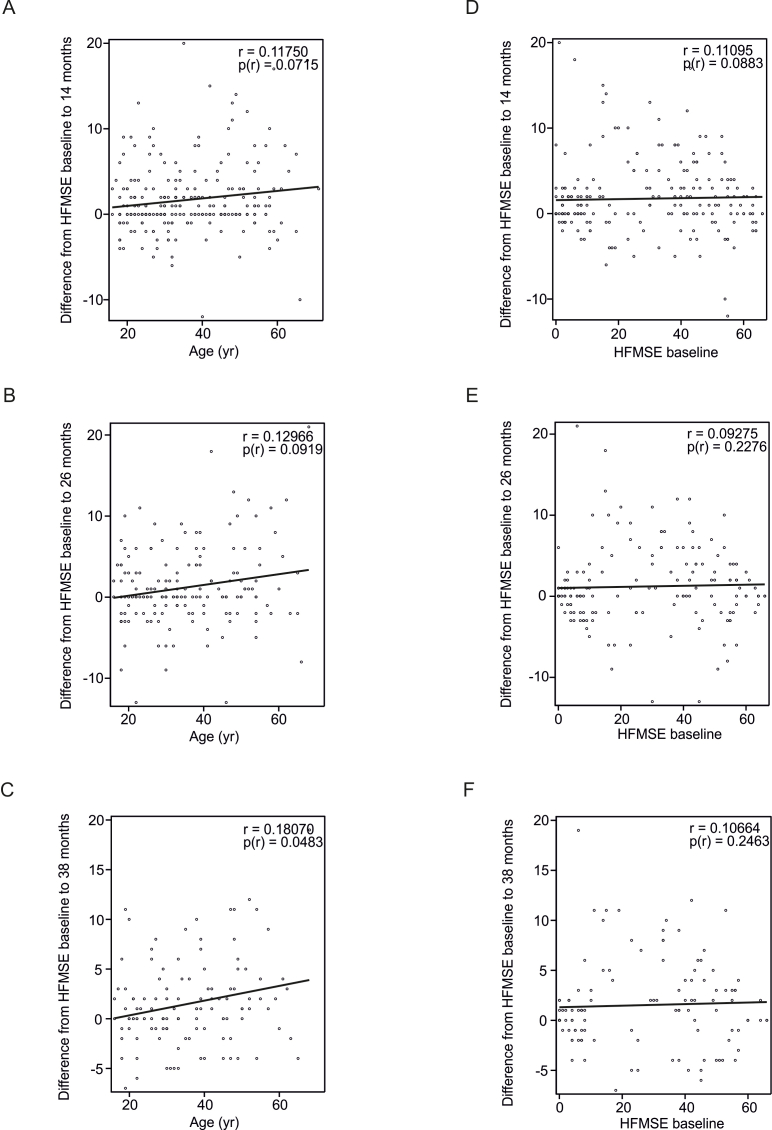
**Supplement Figure 2.** Correlation analysis between the change in HFMSE score (baseline to 14 months (A), baseline to 26 months (B) and baseline to 38 months (C)) and the patient`s age. Correlation analysis between the change in HFMSE score (baseline to 14 months (D), baseline to 26 months (E) and baseline to 38 months (F)) and the HFMSE score at baseline. HFMSE = Hammersmith Functional Motor Scale Expanded.

Two patients had a full score (66 points) and 55 had 0 points on the HFMSE at baseline. Compared with baseline, the mean HFMSE scores were significantly higher at 14 months (mean difference 1.72 [95% CI 1.19–2.25]), 26 months (mean difference 1.20 [95% CI 0.48–1.91]), and 38 months (mean difference 1.52 [95% CI 0.74–2.30]) after initiation of treatment with nusinersen (Fig. 2, Table 2). Clinically meaningful improvements (≥3 points) in the HFMSE score were seen in 68 (29%; mean difference 6.66 [95% CI 5.70–7.62]) patients at 14 months, 49 (29%; mean difference 6.86 [95% CI 5.75–7.96]) patients at 26 months, and 36 (30%; mean difference 6.64 [95% CI 5.43–7.85]) patients at 38 months. Twenty-eight patients with clinically meaningful improvement at 14 months maintained the improvement for ≥38 months of treatment. Compared with baseline, worsening of motor function (<0 points) was seen in 47 (20%; mean difference −2.68 [95% CI −3.34 to −2.02]) patients at 14 months, 49 (29%; mean difference −3.53 [95% CI −4.35 to −2.72]) patients at 26 months, and 34 (28%; mean difference −2.88 [95% CI −3.48 to −2.28]) patients at 38 months after treatment with nusinersen. Clinically meaningful worsening (−3 points or more) was observed in 19 (8%; mean difference −4.58 [95% CI −5.76 to −3.39]) of these patients at 14 months, 25 (15%; mean difference −5.32 [95% CI −6.56 to −4.08]) at 26 months, and 16 (13%; mean difference −4.50 [95% CI −5.01 to −3.99]) at 38 months. The further course of the subgroups with improvement (>0 points), without change (=0 points), and with worsening (<0 points) at 14 months compared to baseline are displayed in Supplementary Figure S1A correlation analysis revealed only a negligible correlation between the change in HFMSE score and the patient's age at all three time points of treatment (r < 0.3). No correlation was found between the patient's age and HFMSE score at baseline (r = 0.04, p = 0.4947). No significant associations between HFMSE score at baseline and improvements in the HFMSE scores were found (Supplementary Figure S2). Ambulatory patients showed a negative correlation of baseline HFMSE scores to change of HFMSE at follow-up time points (i.e. 38 months; r = −0.43, p ≤ 0.0001), which has to be interpreted as ceiling effect in patients with high HFMSE scores at baseline.

**Table 2:** Changes in HFMSE, RULM, and 6-MWT scores.

	n	Scores, mean (SD)	n	Difference versus baseline, mean (95% CI)	p-value (paired t-test)	p-value (Wil-coxon signed-rank test)	Clinically meaning-ful improve-ment, n (%)
**14 months**							
HFMSE	237	27.04 (22.28)	237	1.72 (1.19–2.25)	<0.0001	<0.0001	68 (28.7)
RULM	244	24.06 (12.18)	237	0.75 (0.43–1.07)	<0.0001	<0.0001	68 (28.7)
6 MWT	89	372.48 (189.47)	82	30.86 (18.34–43.38)	<0.0001	<0.0001	39 (47.6)
**26 months**							
HFMSE	171	27.47 (22.40)	171	1.20 (0.48–1.92)	0.0012	0.0021	49 (28.7)
RULM	180	24.57 (11.65)	173	0.65 (0.27–1.03)	0.0009	0.0004	48 (27.7)
6 MWT	70	369.51 (198.47)	62	29.26 (14.87–43.65)	0.0001	0.0005	26 (41.9)
**38 months**							
HFMSE	120	27.68 (21.05)	120	1.52 (0.74–2.30)	0.0002	0.0006	36 (30.0)
RULM	128	24.90 (12.27)	123	0.72 (0.25–1.18)	0.0030	0.0008	32 (26.0)
6 MWT	49	354.92 (191.77)	44	32.20 (10.32–54.09)	0.0049	0.0052	21 (47.7)

Maximum score values: HFMSE_max_ = 66; RULM_max_ = 37. 6 MWT, 6-min walk test; CI, confidence interval; HFMSE, Hammersmith Functional Rating Motor Scale Expanded; RULM, Revised Upper Limb Module; SD, standard deviation.

Because of a relevant number of missing values, we substituted missing values (missing values: at 14 months 152/389 (39%), at 26 month 218/389 (56%), at 38 month 269/389 (69%) using the fully conditional specification (FCS) method in an exploratory analysis. By doing so, mean change of HFMSE was 1.43 [95% CI 0.48–2.38] at 14 months, 0.75 [95% CI –0.32 to 1.83] at 26 months, and 0.54 [95% CI –0.46 to 1.55] at 38 months. Mean change difference was only statistically significant at month 14 (p = 0.0032) (Month 26: p = 0.169; Month 38: p = 0.289).

**Fig. 3:**
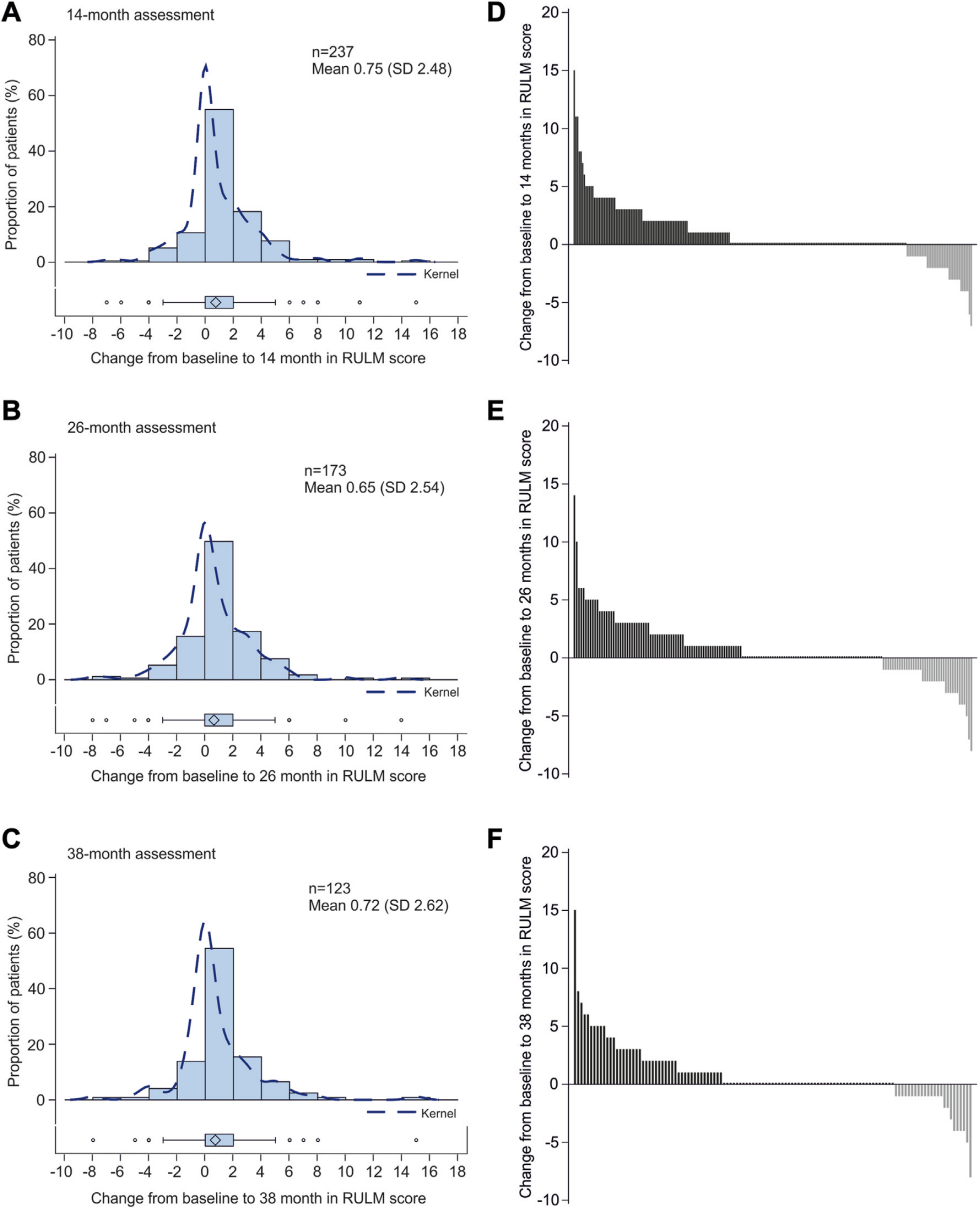
Changes in RULM score from baseline to 14 (A, D), 26 (B, E) and 38 (C, F) months. Left panels show distribution of changes in RULM score from baseline to 14, 26, and 38 months, with each bar representing the proportion of patients who had improved/worsened. The dashed line is the kernel density estimator for the distribution. Box and whisker plots show median (central line), IQR (boxes), and 1.5 × IQR (whiskers), with individual points representing outliers (those outside of 1.5 × IQR from the median). Diamonds indicate the mean values. Panels on the right show changes in RULM in individual patients from baseline. Each bar represents a single patient. RULM = Revised Upper Limb Module; IQR = interquartile range.

**Supplementary Figure S3:**
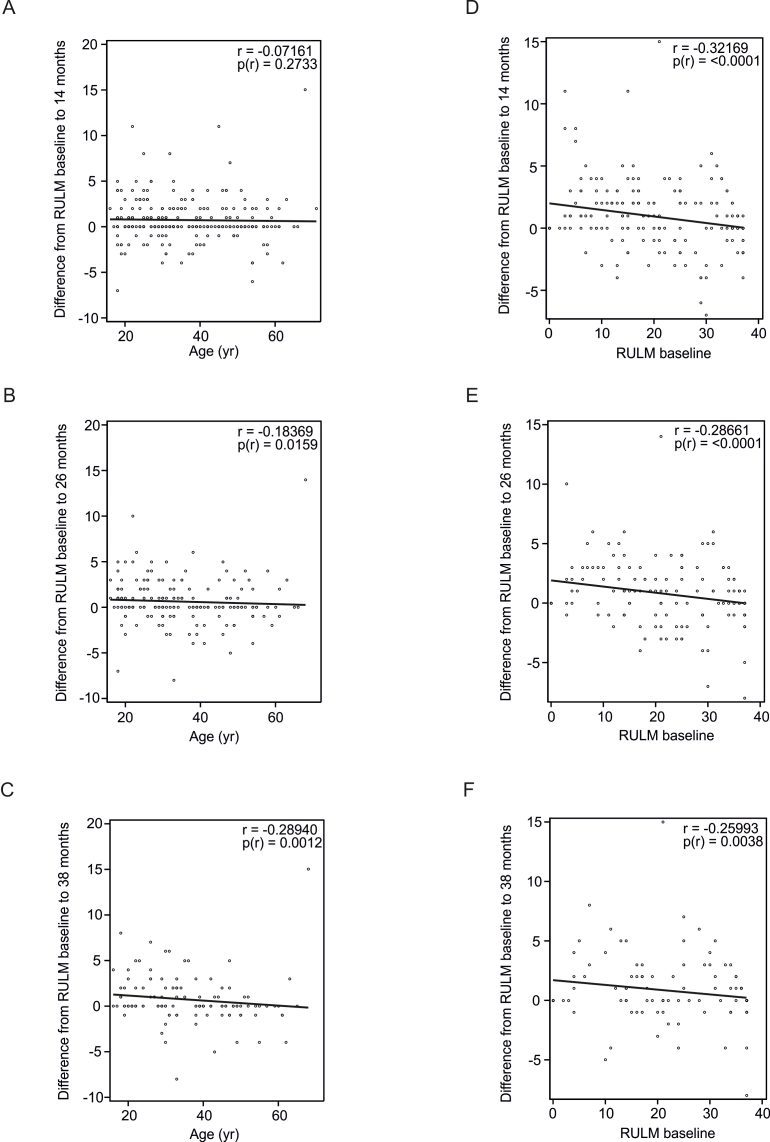
**Supplement figure 3.** Correlation analysis between the change in RULM score (baseline to 14 months (A), baseline to 26 months (B) and baseline to 38 months (C)) and the patient`s age. Correlation analysis between the change in RULM score (baseline to 14 months (D), baseline to 26 months (E) and baseline to 38 months (F)) and the RULM score at baseline. RULM = Revised Upper Limb Module.

Seventy-four patients had a full score (37 points) and 17 patients had 0 points on the RULM at baseline. Compared with baseline, the RULM was also significantly higher at 14 months (mean difference 0.75 [95% CI 0.43–1.07]), 26 months (mean difference 0.65 [95% CI 0.27–1.03]), and 38 months (mean difference 0.72 [95% CI 0.25–1.18]) after initiation of treatment with nusinersen (Fig. 3, Table 2). Clinically meaningful improvements (≥2 points) in the RULM score were seen in 68 (29%; mean difference 3.6 [95% CI 3.02–4.18])) patients at 14 months, 48 (28%; mean difference 3.65 [95% CI 3.01–4.28]) patients at 26 months, and 32 (26%; mean difference 3.91 [95% CI 2.97–4.85]) patients at 38 months (Table 2). Twenty-five (37%) of the 68 patients who showed a clinically meaningful increase in RULM score at 14 months remained stable until 38 months of treatment. Thirty (24%) patients had maintained a full RULM score (37 points) at 38 months after treatment initiation. Thirty-seven (30%) patients showed no clinically meaningful change, and 24 (20%; mean difference −2.13 [95% CI −2.90 to −1.35])) showed a decline of ≥1 points during the 38-month observation period. Compared with baseline, a clinically meaningful worsening (−2 points or more) was seen in 27 patients (11%; mean difference −2.96 [95% CI −3.47 to −2.45]) at 14 months, 22 (13%; mean difference −3.18 [95% CI −3.91 to −2.45]) at 26 months, and nine (7%; mean difference −4.00 [95% CI −5.39 to −2.61]) at 38 months. The further course of the subgroups with improvement (>0 points), without change (=0 points), and with worsening (<0 points) at 14 months compared to baseline are displayed in Supplementary Figure S1B. Correlation analysis showed negligible correlations between age and change in RULM score from baseline to 26 months (r = −0.19, p < 0.05) and to 38 months (r = −0.29, p < 0.01) of treatment. No correlation was found with change in RULM score from baseline to 14 months (r = 0.09, p = 0.1362). A weak negative correlation was found between baseline RULM score and change in RULM score at 14 months (r = −0.32, p < 0.001), 26 months (r = −0.29, p < 0.001), and 38 months (r = −0.26, p < 0.01) (Supplementary Figure S3).

**Supplementary Figure S4:**
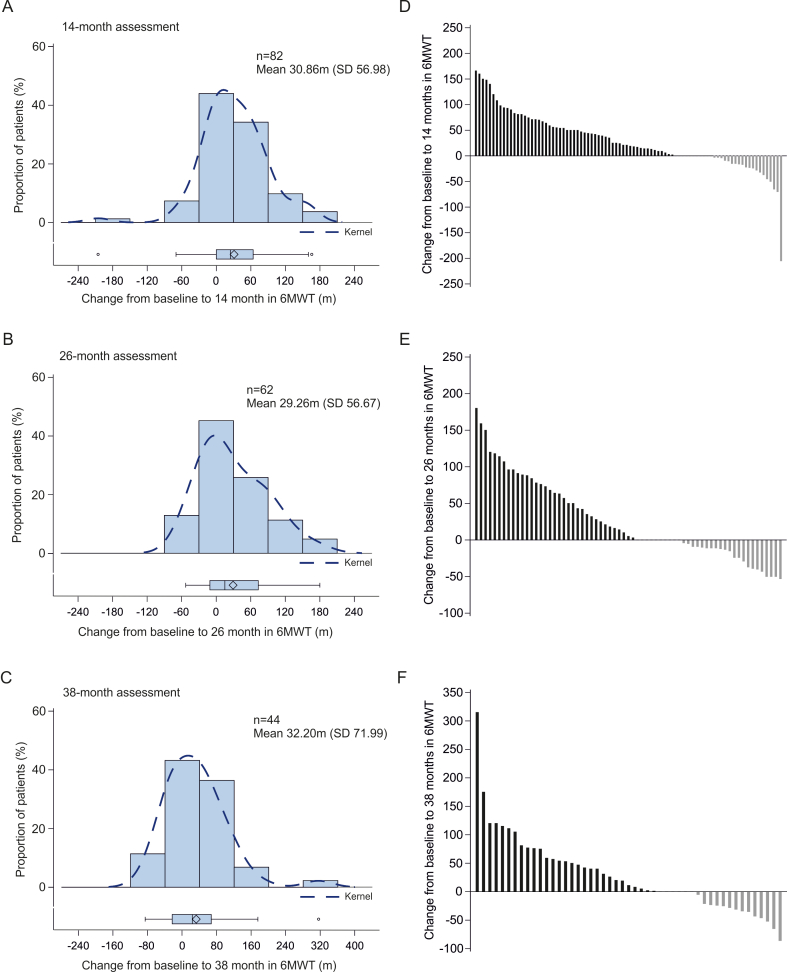
**Supplement figure 4.** Changes in 6MWT (in m) from baseline to 14 (A, D), 26 (B, E), and 38 (C, F) months. Left panels show distribution of changes in 6MWT from baseline to 14, 26, and 38 months, with each bar representing the proportion of patients who had improved/worsened. The dashed line is the kernel density estimator for the distribution. Box and whisker plots show median (central line), IQR (boxes), and 1·5 × IQR (whiskers), with individual points representing outliers (those outside of 1·5 × IQR from the median). Diamonds indicate mean values. Panels on the right show changes in 6MWT in individual patients from baseline. Each bar represents a single patient. IQR = interquartile range; m=metres; 6MWT = six-minute walk test.

Compared with baseline, the mean walking distance in the 6 MWT was significantly longer at 14 months (mean difference 30.86 m [95% CI 18.34–43.38]), 26 months (mean difference 29.26 m [95% CI 14.87–43.65]), and 38 months (mean difference 32.20 m [95% CI 10.32–54.09]) after initiation of treatment with nusinersen (Table 2, Supplementary Figure S4). Clinically meaningful improvements (≥30 m) in 6 MWT were seen in 39 (48%; mean difference 75.83 [95% CI 64.03–87.63]) patients at 14 months, 26 (42%; mean difference 85.50 [95% CI 70.11–100.89]) patients at 26 months, and 21 (48%; mean difference 87.76 [95% CI 58.89–116.63]) patients at 38 months (Table 2). A clinically meaningful worsening (−30 m or more) was observed in seven (9%; mean difference −72.00 [95% CI −127.72 to −16.28]) patients at 14 months, eight (13%; mean difference −45.25 [95% CI −50.42 to −40.08]) patients at 26 months, and eight (18%; mean difference −49.00 [95% CI −64.56 to −33.44]) patients at 38 months.

Exploratory subgroup analysis showed that compared with baseline, the mean HFMSE scores were significantly higher at all three timepoints in patients with SMA type 3, and at 14 months in patients with SMA type 2. No significant changes were observed at 26 and 38 months in SMA type 2. The mean difference in HFMSE vs. baseline was significantly lower in SMA type 2 than type 3 at 26 and 38 months (p < 0.01 at both timepoints) (Supplementary Tables S1 and S2). The mean RULM scores were also significantly higher compared with baseline at all three timepoints in patients with SMA type 2, and at 14 and 38 months in patients with SMA type 3 (Supplementary Table S3). Comparison of SMA type 2 and 3 revealed significantly higher mean differences in RULM scores vs. baseline at 14 and 26 months in SMA type 2 than type 3 (p < 0.01 at both timepoints).

Exploratory subgroup analysis between ambulatory and non-ambulatory patients revealed that HFMSE scores were significantly higher at 14 months vs. baseline in both subgroups. However, at 26 and 38 months the increase was only observed in ambulatory patients. The mean difference in HFMSE scores vs. baseline were significantly lower in non-ambulatory patients compared to ambulatory patients at all three timepoints (p ≤ 0.01 at 14 months; p < 0.01 at 26 months; p < 0.01 at 38 months) (Supplementary Table S2). The RULM scores were significantly higher at all three timepoints compared with baseline in the non-ambulatory subgroup. In contrast, no changes in the ambulatory subgroup were observed (Supplementary Table S3). The mean differences in RULM scores vs. baseline were significantly higher in non-ambulatory patients compared to ambulatory patients at all three timepoints (p < 0.05 at 14 months; p = 0.05 at 26 months; p = 0.02 at 38 months). The HFMSE scores were higher at 14 months than baseline in patients with and without spondylodesis; higher scores at 26 and 38 months compared with baseline were only observed in the group without spondylodesis. The RULM scores were significantly higher at all timepoints in patients with and without spondylodesis (Supplementary Tables S2 and S3). Significant mean differences in HFMSE scores vs. baseline between patients with and without spondylodesis were found at 26 and 38 months; significant differences in the RULM scores between these groups were only observed at 14 months (Supplementary Tables S2 and S3).

Overall, 732 adverse drug reactions or procedure-related complications were documented in 353/389 (91%) screened patients who received at least one injection. Fifty-one (7%) patients had adverse drug reactions without a detailed description. No adverse drug reactions were reported in 103 (26%) patients, while 250 (64%) patients had at least one adverse reaction. There was no hint of more AEs in the group of worsening patients (improving: 75% vs. worsening: 64% ≥ 1 AE). Most adverse reactions were post-lumbar puncture syndrome, headache, backpain, and infections (for details, see Supplementary Table S4).

## Discussion

The therapeutic options for SMA have changed considerably in recent years. Whereas the efficacy of nusinersen in infants and children has been demonstrated in randomised clinical trials,^18^^,^^19^ efficacy in adult patients with SMA with different phenotypes has mainly been documented in real-world evidence studies and only in minority in RCTs.^20^ These have shown that nusinersen may improve or stabilise motor function over a period of up to 24 months, a finding that has been confirmed by several meta-analyses.^12^, ^13^, ^14^^,^^21^ Longer treatment periods have not yet been investigated. Our study demonstrates that nusinersen leads to long-lasting improvement or stabilisation in motor function for up to 38 months in the majority of adult SMA patients, while in almost 30% a worsening in motor scale assessments was observed. The tolerability was good and not different between patients with improvement or worsening in motor scale assessments. This is the largest study in adult patients with SMA reported to date and reflects the broad heterogeneity in this population, including ambulatory and non-ambulatory patients over a wide age range.

Our results show that in less severely affected patients in particular (i.e. SMA type 3, ambulatory and without spondylodesis), a relevant number of patients experience a sustained and clinically relevant improvement in motor function. With increasing disease severity (i.e. SMA type 2, non-ambulatory and with spondylodesis), the proportion of patients with a clinically relevant improvement is smaller; however, we did observe sustained stabilisation of symptoms. Although our study did not include patients that represented the natural history of the disease, previous studies of disease progression indicate that a relevant deterioration in motor function could have been expected over the observation time period of 38 months.^22^ A recent study, including mostly SMA type 2 and 3 patients, on the natural history of SMA reported an estimated decline of 0.5 points in the HFMSE score per year.^3^ The dynamic in the RULM score of untreated patients with SMA type 2 was recently described as a loss of 0.8 points over 12 month and 1.52 points over 24 month. Non ambulatory SMA type 3 patients also had a decline of 0.9 points in RULM score, whereas ambulatory patients did not show changes.^23^ In ambulatory patients the distance in the 6 MWT decreased 9.7 m in distance^24^ Thus, stabilisation of the otherwise progressive disease course in severely affected patients can be interpreted as efficacy.

It should be noted that in very severely affected patients, as well as patients with mild motor impairment, use of HFMSE and RULM as measuring instruments does not necessarily reflect individual improvements due to ceiling and floor effects.^25^ The limitations are in the assessment of distal motor function, which is of utmost importance for severely affected patients with SMA, as well as gross motor function in more mildly affected patients.^26^ It has been reported previously, that HFMSE is a more sensitive measure in less severely affected patients, whereas RULM is more sensitive in more severely affected patients and therefore has been suggested to include them as a composite measure.^27^^,^^28^ Our results suggest that this also applies to adult SMA patients.^29^^,^^30^ Compared with the 14-month observation period in our previous study,^11^ the absolute motor improvement at the same time point in the total group of patients in our current study was lower, although significant functional improvement was still detectable. One of the strongest predictors for the extent of improvement in our previous study was the motor function level at baseline.^11^ These earlier results demonstrated that patients with the longest observation period were on average less severely affected at the beginning of therapy than patients with a shorter therapy period.^11^ This might be due to purely practical considerations, as most centres had started therapy with less severely affected patients, and thus less complex spinal anatomy. In the current expanded cohort, which included a larger number of patients with SMA type 2 and thus more severely affected patients, this effect levelled out. Nevertheless, the exploratory analyses continue to consistently show that lower disease severity is associated with greater improvement. As no biomarker of therapeutic response to nusinersen has yet been validated for adult patients in clinical routine, clinical scales remain the most relevant outcome parameter to date.^26^^,^^31^ However, the motor scales we used are unidimensional and focus on gross (HFMSE), upper limb (RULM) motor and ambulatory (6 MWT) function, particularly proximal muscle groups. Since adult patients with SMA show a broadly diversified phenotype, the validity of these scales is limited, especially in the borderline range.^32^ This means that HFMSE score interpretation is limited by floor effects in severely disabled patients (SMA type 2 and non-ambulatory SMA type 3) and RULM score interpretation is limited by ceiling effects in less impaired patients (ambulatory SMA type 3), which may have affected the results of the study. This emphasises that phenotypically individual primary outcome parameters should be defined for the assessment of motor function.

Adverse drug reactions or procedure-related complications were documented in nearly all patients (91%). But adverse event recording did not identify any unusual or new safety signals. Cases of hydrocephalus were reported shortly after approval, including in a few adult patients. In addition, serial cerebrospinal fluid analyses during therapy showed disruption of the blood–brain barrier in individual patients.^33^ In our study, however, there were no clinical signs of these complications in a large number of patients over the course of several years of therapy. Therefore, therapy with nusinersen appears to be safe in the long term.

To date, this is the largest prospective study over the longest observational period of nusinersen therapy in adult patients with SMA. Our results demonstrate sustained efficacy across the phenotypic spectrum of adult SMA, showing stabilisation or improvement in motor function in a large number of patients, which is clearly different from the natural history of the disease.^3^^,^^24^^,^^34^ By using the SMArtCARE registry dataset, consistent standards of care can be assumed because all data came from experienced centres with specifically trained staff. However, there are some limitations. For example, the use of uniform scales in all patients makes it difficult to capture small motor function changes that may be highly relevant for the patients. In addition, no statement can be made about the limitations of the muscles required for swallowing and respiratory function, which may be impaired in severely affected patients. By using phenotype-specific scales, this may be possible in the future. Furthermore, our study does not include a comparison with an untreated cohort. This real-world evidence provides an elaborate and valuable assessment of motor function during nusinersen treatment, even though does not have the same class of evidence as a randomised controlled trial.

## Contributors

TH and RG wrote the manuscript, TH, CDW and RG planned the study, interpreted the data and coordinated the study, and were responsible for local data collection. AO, OSK, SP, ZU, MH, ST, MCW, MWei, TK, MF, HSL, IC, PL, MD, AHa, KM, RS, BI, AR, JB, JN, DZ, MTM, TB, JCK, BS, SF, AHe, CK, SN, AM, MWeb, CN, HCL, GW, CS, MT, AG, MG, MT, MS, GB, PM, CR, GMzH, PB, WL, MFB, CK, KP, SG, US, WMF, HL, SMArtCare study group, CK, ACL were responsible for local data collection and contribution and critically revised the manuscript. OvV was responsible for data analysis, figure production, and writing of the statistical part of the manuscript. AP and JK are responsible for database preparation and data processing of the SMArtCARE registry and were involved in the study design as well as critical revision of the manuscript. TH, RG, OvV and JK has directly accessed and verified the underlying data reported in the manuscript. Deborah Nock (Medical WriteAway, Norwich, UK) reviewed the English language in the draft manuscript, funded by Essen University Hospital.

## Data sharing statement

Data can be obtained anonymized and aggregated upon request and approval by the authors and the SMArtCARE steering committee.

## Declaration of interests

SB, ZU, MH, TK, KM, BI, JB, MTM, TB, BS, SF, CS, MTo, AG, MTue, MS, CR, PB, MFB, CK, KPS, SG, ST, JN, RS, MWeb, GW and OvV declare no conflicts of interest.

RG has received personal fees from Biogen and Hoffmann-La Roche and served on advisory boards from Biogen, Hoffmann-La Roche, ITF Pharma, Zambon and research support from Biogen, outside of the submitted work.

CDW has received personal fees from Biogen and Hoffmann–La Roche outside of the submitted work. AO has received speaker fees from Biogen outside of the submitted work.

OSK received academic research support from the Hannover Medical School (MHH) and the German Neuromuscular Society “Deutsche Gesellschaft fuer Muskelkranke” (DGM e.V.), 2019–2021 (grant no. Sc 23/1); and received honoraria as a speaker and/or funding for travel expenses from the German Neuromuscular Society “Deutsche Gesellschaft fuer Muskelkranke (DGM e.V.), Biogen GmbH, Biermann Verlag GmbH, and MK + S—Medizin, Kommunikation & Service GmbH, outside the submitted work.

SP has received speaker fees, non-financial support and research support from Biogen, Roche, AL-S Pharma, Amylyx, Cytokinetics, Ferrer, ITF-Pharma, Zambon, and Sanofi and served on advisory boards of Amylyx, Biogen, Roche, Zambon and ITF Pharma outside of the submitted work.

MCW has served on advisory boards for Avexis, Biogen, Grünenthal, Novartis, Pfizer, PharNext, PTC Therapeutics, Roche, Santhera, Sarepta, Ultragenyx, Wave Sciences, received funding for Travel or Speaker Honoraria from Biogen, Novartis, PTC Therapeutics, Santhera, and worked as an ad-hoc consultant for Affinia, Audentes Therapeutics, Avexis, Biogen, BridgeBio, Edgewise, Fulcrum, Grünenthal, ML Bio, Novartis, Pfizer, PharNEXT, PTC Therapeutics, Roche.

MWei has received advisory board and consultant honoraria from Biogen and Hoffmann-La Roche, and speaker honoraria and travel support for conference attendance from Biogen, outside of the submitted work. MW is a member of the European Reference Network for Neuromuscular Diseases (ERN EURO-NMD).

MF has received a speaker honorarium and non-financial support from Biogen outside the submitted work.

HSL is receiving advisory fees from Biogen but has no financial or non-financial conflict of interest to declare related to the content of this manuscript.

IC has received research grants and speaker fees from Biogen and Hoffmann-La Roche, outside of the submitted work.

PL has received honoraria for advisory boards and consultancies from Stadapharm, Abbvie, Alexion, Bial, ITF Pharma, Desitin, Novartis, Woolsey Pharma outside the scope of this work.

MD has received personal fees as speaker/consultant from Biogen and Roche, outside of the submitted work.

Aha received research grants from Novartis Gene Therapies, and advisory board honoraria and speaker fees from Biogen, Roche, and Novartis.

AR has received advisory board honoraria from Biogen outside of the submitted work.

DZ received compensation from Biogen for participation on advisory boards, from Novartis for consultancy work, and travel compensation from Angelini Pharma outside of the submitted work.

JCK has received personal fees from Biogen and Roche for advisory boards and development of educational material outside of this study.

AHe has received personal fees and non-financial support from Biogen and Desitin for advisory board meetings outside the reported work.

CK has received advisory board honoraria from Biogen, Roche and Ipsen Pharma, speaker honoraria from Biogen and unrestricted travel grants from Ipsen outside of the submitted work.

SN has received financial support for consultancy and lecturing from Allergan, Hormosan, Lilly, Lundbeck, Novartis, Teva and Medscape, research support from Novartis, all outside of the submitted work.

AM has received advisory board honoraria from Hormosan and Sanofi, outside of the submitted work.

CN has received personal fees from Biogen and Hoffmann–La Roche outside of the submitted work.

HCL received honoraria for speaking and advisory board engagements or academic research support Biogen.

MG has received an advisory board honorarium from Hoffmann-La Roche and a speaker fee from Novartis outside of the submitted work.

AP received compensation for advisory boards, training activities and research grants from Novartis and Biogen.

JK received compensation for clinical research and/or consultancy activities from Biogen, Novartis, Roche and ScholarRock.

GB has received research grants from PTC, advisory board honoraria and speaker fees from Biogen, Hoffmann-La Roche, Novartis, Pfizer, PTC and personal fees from Roche outside of the submitted work.

PM has received honorary as an advisory board member from Biogen unrelated to this work.

GMzH received compensation for serving on scientific advisory boards (Alexion, Roche, LFB) and speaker honoraria (Alexion).

WL received advisory board honoraria and speaker fees from Biogen and Roche outside of the submitted work MFB has received honoraria from Biogen, Roche and Novartis as an advisory board member and for lectures from Novartis.

US has received honoraria for counseling at advisory boards and invited talks for Biogen, Novartis and Roche.

WMF has received compensation for scientific advisory boards for Biogen, Novartis, PTC, Sarepta, Sanofi-Aventis, Roche and Cytokinetics and received travel expenses and speaker fees from Biogen, Novartis, PTC, Roche, Sarepta and Sanofi-Aventis.

HLo received support for research projects and clinical trials from Amplo Biotechnology, AMO Pharma, argenx, Biogen, Desitin, Fulcrum Therapeutics, Harmony Biosciences, KYE Pharmaceuticals, Milo Biotechnology, Novartis, Pfizer, PTC Therapeutics, Hoffman-La Roche Limited, Sanofi-Genzyme, Santhera, Sarepta, Satellos, Spark Therapeutics and Ultragenyx. HL is the Editor-in-chief for the Journal of Neuromuscular Diseases (IOS Press).

CK has received compensation for lectures and advisory boards as well as research funds from Biogen, Roche and Novartis.

ACL is a member of Advisory Boards of Roche Pharma AG, Biogen, Alector and Amylyx. He received compensation for talks from Biologix, the German Society of Neurology, Biogen, Springer Medicine, Amylyx and the company Streamed Up! GmbH. He is involved in trials which are sponsored by Amylyx, Ferrer International, Novartis Research and Development, Mitsubishi Tanabe, Apellis Pharmaceuticals, Alexion, Orion Pharma, the European Union, BMBF, Biogen and Orphazyme, Ionis Pharmaceuticals, QurAlis and Alector.

TH has received research grants, advisory board honoraria and speaker fees from Biogen, Hoffmann-La Roche, Novartis and personal fees from Roche and Novartis outside of the submitted work.
