# Supplementary material for: Long-term efficacy and safety of nusinersen in adults with 5q spinal muscular atrophy: a prospective European multinational observational study
Source: Lancet Reg Health Eur. 2024 Feb 6;39:100862. doi: 10.1016/j.lanepe.2024.100862 (PMC10864329; doi:10.1016/j.lanepe.2024.100862)
Supplement: Supplementary Tables S1–S4 [file mmc1.docx]

Supplement table 1: Result of multivariate Analysis: Estimates and Confidence Limits of Fixed Effects; Dependent Variable: HFMSE (in Score)

Mixed Model Analyse

| **Parameter** | **Estimate** | **Standard Error** | **95% Confidence**  **Interval** | | **p Value** |
| --- | --- | --- | --- | --- | --- |
| **Intercept** | 10.6031 | 1.9862 | 6.6929 | 14.5133 | <0.0001 |
| **Sex**  male | -3.3862 | 1.2407 | -5.8239 | -0.9486 | 0.0066 |
| **Age** | -0.2893 | 0.05194 | -0.3914 | -0.1873 | <0.0001 |
| **SMA Type**  type=3 | 11.1536 | 1.5810 | 8.0473 | 14.2599 | <0.0001 |
| **Ambulant**  yes | 31.3375 | 1.4439 | 28.5005 | 34.1745 | <0.0001 |
| **Spondylodesis** no | 6.7567 | 1.8122 | 3.1961 | 10.3173 | 0.0002 |
| **Follow-up*** |  |  |  |  |  |
| month 14 | 1.7040 | 0.2552 | 1.2026 | 2.2055 | <0.0001 |
| month 26 | 1.0063 | 0.2899 | 0.4366 | 1.5759 | 0.0006 |
| month 38 | 1.1484 | 0.3278 | 0.5044 | 1.7924 | 0.0005 |

*The estimates for the HFMSE scores at months 14, 26 and 38 using baseline HFMSE as reference

# Supplement table 2: Differences in HFMSE Score (versus baseline)

| HFMSE Score | Follow-up | n | **Mean difference versus baseline (SD; 95% CI)** | p value (paired t-Test) | p value (Mann- Whitney-U test) for group differences | Clinically meaningful improvement **n (%)** |
| --- | --- | --- | --- | --- | --- | --- |
| SMA type |  |  |  |  |  |  |
| 2 | 14-month | 67 | 1.0 (3.1 -0.2–1.7) | 0.0099 |  | 9 (13.2%) |
|  | 26-month | 44 | -0.5 (3.3; -1.5–0.5) | 0.3400 |  | 3 (6.8%) |
|  | 38-month | 33 | -0.2 (2.8; -1.2–0.8) | 0.6704 |  | 2 (6.1%) |
| 3 | 14-month | 156 | 2.0 (4.4; 1.3–2.7) | <0.0001 | 0.0542 | 54 (34.6%) |
|  | 26-month | 117 | 1.7 (4.8; 0.9–2.6) | 0.0002 | 0.0077 | 44 (37.6%) |
|  | 38-month | 83 | 2.3 (4.6; 1.2–3.3) | <0.0001 | 0.0023 | 33 (39.8%) |
| Ambulant |  |  |  |  |  |  |
| yes | 14-month | 100 | 2.5 (4.8; 1.6–3.5) | <0.0001 |  | 44 (44.0%) |
|  | 26-month | 77 | 2.2 (5.2; 1.0–3.4) | 0.0004 |  | 33 (42.9%) |
|  | 38-month | 57 | 2.4 (4.6; 1.7–3.6) | 0.0002 |  | 26 (45.6%) |
| no | 14-month | 137 | 1.1 (3.5; 0.5–1.7) | 0.0003 | 0.0026 | 24 (17.5%) |
|  | 26-month | 94 | 0.4 (4.1; -0.5–1.2) | 0.3991 | 0.0032 | 16 (17.0%) |
|  | 38-month | 63 | 0.7 (3.9; -0.3–1.7) | 0.1439 | 0.0091 | 10 (15.9%) |
| Spondylodesis |  |  |  |  |  |  |
| yes | 14-month | 45 | 0.8 (2.2; 0.2–1.5) | 0.0121 |  | 5 (11.1%) |
|  | 26-month | 40 | -0.5 (2.2; -1.2–0.2) | 0.1722 |  | 1 (2.5%) |
|  | 38-month | 26 | -0.3 (1.6; -0.9–0.4) | 0.3883 |  | 0 (0.0%) |
| no | 14-month | 192 | 1.9 (4.5; 1.3–2.6) | <0.0001 | 0.2379 | 63 (32.8%) |
|  | 26-month | 131 | 1.7 (5.2; 0.8–2.6) | 0.0002 | 0.0031 | 48 (36.6%) |
|  | 38-month | 94 | 2.0 (4.7; 1.1–3.0) | <0.0001 | 0.0082 | 36 (38.3%) |

Maximum values of HFMSE_max_ = 66, SD, standard deviation; HFMSE, Hammersmith Functional Rating Motor Scale Expanded

#

# Supplement table 3: Differences in RULM Score (versus baseline)

| RULM Score | Follow-up | n | **Mean difference versus baseline (SD; 95% CI)** | p value (paired t -test) | p value (Mann- Whitney-U test) for group differences | Clinically meaningful improvement **n (%)** |
| --- | --- | --- | --- | --- | --- | --- |
| SMA type |  |  |  |  |  |  |
| 2 | 14-month | 68 | 1.4 (2.8; 0.7–2.1) | 0.0001 |  | 29 (42.6%) |
|  | 26-month | 45 | 1.4 (2.6; 0.6–2.1) | 0.0009 |  | 17 (37.8%) |
|  | 38-month | 32 | 1.1 (2.7; 0.1–2.1) | 0.0298 |  | 12 (37.5%) |
| 3 | 14-month | 155 | 0.4 (2.3; 0.2–0.9) | 0.0059 | 0.0026 | 36 (23.2%) |
|  | 26-month | 119 | 0.4 (2.6; -0.07–0.9) | 0.0978 | 0.0093 | 29 (24.4%) |
|  | 38-month | 86 | 0.6 (2.6; 0.0–1.2) | 0.0332 | 0.2415 | 18 (20.9%) |
| Ambulant |  |  |  |  |  |  |
| yes | 14-month | 98 | 0.3 (1.6; -0.01–0.6) | 0.0593 |  | 16 (16.3%) |
|  | 26-month | 74 | 0.2 (1.9; -0.3–0.6) | 0.4639 |  | 10 (13.5%) |
|  | 38-month | 57 | 0.1 (2.0; -0.4–0.7) | 0.6448 |  | 8 (14.0%) |
| no | 14-month | 139 | 1.1(2.9; 0.5–1.5) | <0.0001 | 0.0113 | 52 (37.4%) |
|  | 26-month | 99 | 1.0 (2.9; 0.3–1.6) | 0.0007 | 0.0515 | 38 (38.4%) |
|  | 38-month | 66 | 1.2 (3.0; 0.5–2.0) | 0.0013 | 0.0205 | 24 (36.4%) |
| Spondylodesis |  |  |  |  |  |  |
| yes | 14-month | 44 | 1.7 (3.1; 0.8–2.6) | 0.0007 |  | 22 (50.0%) |
|  | 26-month | 41 | 1.0 (2.7; 0.2–1.9) | 0.0192 |  | 15 (36.6%) |
|  | 38-month | 26 | 1.0 (2.2; 1.1–1.9) | 0.0248 |  | 11 (42.3%) |
| no | 14-month | 193 | 0.5 (2.3; 0.2–0.9) | 0.0013 | 0.0009 | 46 (23.8%) |
|  | 26-month | 132 | 0.5 (2.5; 0.1–1.0) | 0.0144 | 0.1399 | 33 (25.0%) |
|  | 38-month | 97 | 0.6(2.7; 0.1–1.2) | 0.0248 | 0.1675 | 20 (20.6%) |

Maximum values of RULM_max_ = 37; SD, standard deviation; RULM, Revised Upper Limb Module

# Supplement table 4: Adverse events in adult SMA patients treated with nusinersen

| **Type of adverse event** | **MedDRA code (PT Term)** | **Number of adverse event related to**  **patients**  **(in total 389)** | **Number of adverse events including repetitions**  **(in total 732)** |
| --- | --- | --- | --- |
| adverse event = true, however no description available | - | 39 (10%) | 51 (7%) |
| adverse_event_type/lumbar | Post lumbar puncture syndrome | 85 (21.9%) | 145 (19.8%) |
|  | Headache | 61 (15.7%) | 100 (13.7%) |
|  | Back pain | 14 (3.6%) | 15 (2%) |
|  | Dizziness | 2 (0.5%) | 2 (0.3%) |
|  | Pain in extremity | 2 (0.5%) | 2 (0.3%) |
|  | Fatigue | 1 (0.3%) | 1 (0.1%) |
|  | Flushing | 1 (0.3%) | 1 (0.1%) |
|  | Injection site discomfort | 1 (0.3%) | 1 (0.1%) |
|  | Injection site pain | 1 (0.3%) | 1 (0.1%) |
|  | Nausea | 1 (0.3%) | 1 (0.1%) |
|  | Neck pain | 1 (0.3%) | 1 (0.1%) |
|  | Palpitations | 1 (0.3%) | 1 (0.1%) |
|  | Procedural pain | 1 (0.3%) | 1 (0.1%) |
|  | Puncture site pain | 1 (0.3%) | 1 (0.1%) |
|  | Sciatica | 1 (0.3%) | 1 (0.1%) |
|  | Spinal pain | 1 (0.3%) | 1 (0.1%) |
|  | Tongue spasm | 1 (0.3%) | 1 (0.1%) |
|  | Vomiting | 1 (0.3%) | 1 (0.1%) |
| adverse_event_type/neuro | Dorsal root ganglion block | 2 (0.5%) | 2 (0.3%) |
| adverse_event_type/other | Back pain | 33 (8.5%) | 48 (6.6%) |
|  | Headache | 32 (8.2%) | 42 (5.7%) |
|  | Fall | 12 (3.1%) | 18 (2.5%) |
|  | Arthralgia | 9 (2.3%) | 9 (1.2%) |
|  | Musculoskeletal discomfort | 8 (2.1%) | 11 (1.5%) |
|  | Diarrhoea | 6 (1.5%) | 6 (0.8%) |
|  | Pain in extremity | 6 (1.5%) | 6 (0.8%) |
|  | Pneumonia | 6 (1.5%) | 7 (1%) |
|  | Constipation | 5 (1.3%) | 5 (0.7%) |
|  | Dizziness | 5 (1.3%) | 5 (0.7%) |
|  | Hypoaesthesia | 5 (1.3%) | 5 (0.7%) |
|  | Nausea | 5 (1.3%) | 7 (1%) |
|  | Ankle fracture | 4 (1%) | 4 (0.5%) |
|  | Sciatica | 4 (1%) | 4 (0.5%) |
|  | Urinary tract infection | 4 (1%) | 5 (0.7%) |
|  | Abdominal pain upper | 3 (0.8%) | 3 (0.4%) |
|  | Circulatory collapse | 3 (0.8%) | 3 (0.4%) |
|  | Erysipelas | 3 (0.8%) | 5 (0.7%) |
|  | Infection via vaccinee | 3 (0.8%) | 3 (0.4%) |
|  | Migraine with aura | 3 (0.8%) | 3 (0.4%) |
|  | Nasopharyngitis | 3 (0.8%) | 3 (0.4%) |
|  | Patella fracture | 3 (0.8%) | 3 (0.4%) |
|  | Pyrexia | 3 (0.8%) | 3 (0.4%) |
|  | Tibia fracture | 3 (0.8%) | 3 (0.4%) |
|  | Abdominal pain | 2 (0.5%) | 2 (0.3%) |
|  | Coronavirus test positive | 2 (0.5%) | 2 (0.3%) |
|  | Diverticulitis | 2 (0.5%) | 2 (0.3%) |
|  | Epistaxis | 2 (0.5%) | 2 (0.3%) |
|  | Gastrointestinal infection | 2 (0.5%) | 2 (0.3%) |
|  | Humerus fracture | 2 (0.5%) | 2 (0.3%) |
|  | Hypertonia | 2 (0.5%) | 3 (0.4%) |
|  | Hypokalaemia | 2 (0.5%) | 2 (0.3%) |
|  | Injection site pain | 2 (0.5%) | 2 (0.3%) |
|  | Muscle spasms | 2 (0.5%) | 2 (0.3%) |
|  | Myalgia | 2 (0.5%) | 3 (0.4%) |
|  | Paraesthesia | 2 (0.5%) | 2 (0.3%) |
|  | Tinnitus | 2 (0.5%) | 2 (0.3%) |
|  | Vertigo | 2 (0.5%) | 3 (0.4%) |
|  | Vomiting | 2 (0.5%) | 2 (0.3%) |
|  | Abdominal pain lower | 1 (0.3%) | 1 (0.1%) |
|  | Abortion | 1 (0.3%) | 1 (0.1%) |
|  | Accident | 1 (0.3%) | 1 (0.1%) |
|  | Acute coronary syndrome | 1 (0.3%) | 1 (0.1%) |
|  | Acute myocardial infarction | 1 (0.3%) | 1 (0.1%) |
|  | Acute sinusitis | 1 (0.3%) | 1 (0.1%) |
|  | Adenoidal hypertrophy | 1 (0.3%) | 1 (0.1%) |
|  | Anaphylactic reaction | 1 (0.3%) | 1 (0.1%) |
|  | Aphthous ulcer | 1 (0.3%) | 1 (0.1%) |
|  | Asthma | 1 (0.3%) | 1 (0.1%) |
|  | Benign neoplasm of skin | 1 (0.3%) | 2 (0.3%) |
|  | Biliary tract disorder | 1 (0.3%) | 1 (0.1%) |
|  | Bone contusion | 1 (0.3%) | 1 (0.1%) |
|  | Borrelia infection | 1 (0.3%) | 1 (0.1%) |
|  | Broad ligament tear | 1 (0.3%) | 1 (0.1%) |
|  | CSF protein increased | 1 (0.3%) | 10 (1.4%) |
|  | Cerebral ischaemia | 1 (0.3%) | 1 (0.1%) |
|  | Cerumen impaction | 1 (0.3%) | 1 (0.1%) |
|  | Choking | 1 (0.3%) | 1 (0.1%) |
|  | Cholecystitis acute | 1 (0.3%) | 1 (0.1%) |
|  | Complicated fracture | 1 (0.3%) | 1 (0.1%) |
|  | Conjunctivitis | 1 (0.3%) | 1 (0.1%) |
|  | Contusion | 1 (0.3%) | 1 (0.1%) |
|  | Cough | 1 (0.3%) | 1 (0.1%) |
|  | Cystitis | 1 (0.3%) | 1 (0.1%) |
|  | Decreased appetite | 1 (0.3%) | 1 (0.1%) |
|  | Decubitus ulcer | 1 (0.3%) | 1 (0.1%) |
|  | Deep vein thrombosis | 1 (0.3%) | 1 (0.1%) |
|  | Dermatitis allergic | 1 (0.3%) | 1 (0.1%) |
|  | Dermatitis contact | 1 (0.3%) | 1 (0.1%) |
|  | Diplopia | 1 (0.3%) | 1 (0.1%) |
|  | Dislocation of vertebra | 1 (0.3%) | 1 (0.1%) |
|  | Dizziness exertional | 1 (0.3%) | 1 (0.1%) |
|  | Dry skin | 1 (0.3%) | 1 (0.1%) |
|  | Dyspepsia | 1 (0.3%) | 1 (0.1%) |
|  | Dyspnoea | 1 (0.3%) | 1 (0.1%) |
|  | Enterocolitis infectious | 1 (0.3%) | 1 (0.1%) |
|  | Eructation | 1 (0.3%) | 1 (0.1%) |
|  | Essential hypertension | 1 (0.3%) | 1 (0.1%) |
|  | Eye infection | 1 (0.3%) | 1 (0.1%) |
|  | Eye irritation | 1 (0.3%) | 1 (0.1%) |
|  | Eye movement disorder | 1 (0.3%) | 1 (0.1%) |
|  | Femoral neck fracture | 1 (0.3%) | 1 (0.1%) |
|  | Femur fracture | 1 (0.3%) | 1 (0.1%) |
|  | Fibula fracture | 1 (0.3%) | 1 (0.1%) |
|  | Foot fracture | 1 (0.3%) | 1 (0.1%) |
|  | Gallbladder operation | 1 (0.3%) | 1 (0.1%) |
|  | Gastroenteritis | 1 (0.3%) | 1 (0.1%) |
|  | Gastroenteritis norovirus | 1 (0.3%) | 1 (0.1%) |
|  | Haemorrhage | 1 (0.3%) | 1 (0.1%) |
|  | Hand fracture | 1 (0.3%) | 1 (0.1%) |
|  | Heat illness | 1 (0.3%) | 1 (0.1%) |
|  | Herpes zoster | 1 (0.3%) | 1 (0.1%) |
|  | Hip fracture | 1 (0.3%) | 1 (0.1%) |
|  | Hot flush | 1 (0.3%) | 1 (0.1%) |
|  | Hypersensitivity | 1 (0.3%) | 1 (0.1%) |
|  | Hyperthermia | 1 (0.3%) | 1 (0.1%) |
|  | Impaired healing | 1 (0.3%) | 1 (0.1%) |
|  | Infection | 1 (0.3%) | 1 (0.1%) |
|  | Influenza | 1 (0.3%) | 1 (0.1%) |
|  | Injection site erythema | 1 (0.3%) | 1 (0.1%) |
|  | Intervertebral disc protrusion | 1 (0.3%) | 1 (0.1%) |
|  | Iron deficiency anaemia | 1 (0.3%) | 1 (0.1%) |
|  | Kidney infection | 1 (0.3%) | 1 (0.1%) |
|  | Laryngitis | 1 (0.3%) | 1 (0.1%) |
|  | Ligament injury | 1 (0.3%) | 1 (0.1%) |
|  | Lower limb fracture | 1 (0.3%) | 1 (0.1%) |
|  | Meningitis aseptic | 1 (0.3%) | 1 (0.1%) |
|  | Meningitis bacterial | 1 (0.3%) | 1 (0.1%) |
|  | Micturition urgency | 1 (0.3%) | 1 (0.1%) |
|  | Mouth haemorrhage | 1 (0.3%) | 1 (0.1%) |
|  | Mouth ulceration | 1 (0.3%) | 1 (0.1%) |
|  | Muscle rupture | 1 (0.3%) | 1 (0.1%) |
|  | Muscle twitching | 1 (0.3%) | 2 (0.3%) |
|  | Nephrolithiasis | 1 (0.3%) | 1 (0.1%) |
|  | Nervous system disorder | 1 (0.3%) | 1 (0.1%) |
|  | Oral herpes | 1 (0.3%) | 1 (0.1%) |
|  | Orchitis | 1 (0.3%) | 1 (0.1%) |
|  | Oscillopsia | 1 (0.3%) | 1 (0.1%) |
|  | PO2 decreased | 1 (0.3%) | 1 (0.1%) |
|  | Palpitations | 1 (0.3%) | 1 (0.1%) |
|  | Paresis cranial nerve | 1 (0.3%) | 1 (0.1%) |
|  | Penile abscess | 1 (0.3%) | 1 (0.1%) |
|  | Peripheral embolism | 1 (0.3%) | 1 (0.1%) |
|  | Post vaccination syndrome | 1 (0.3%) | 1 (0.1%) |
|  | Postoperative wound complication | 1 (0.3%) | 1 (0.1%) |
|  | Procedural pain | 1 (0.3%) | 1 (0.1%) |
|  | Pruritus | 1 (0.3%) | 1 (0.1%) |
|  | Rectal haemorrhage | 1 (0.3%) | 1 (0.1%) |
|  | Respiratory tract infection | 1 (0.3%) | 1 (0.1%) |
|  | Restlessness | 1 (0.3%) | 1 (0.1%) |
|  | Skin irritation | 1 (0.3%) | 2 (0.3%) |
|  | Sleep apnoea syndrome | 1 (0.3%) | 1 (0.1%) |
|  | Spinal fusion surgery | 1 (0.3%) | 1 (0.1%) |
|  | Syncope | 1 (0.3%) | 1 (0.1%) |
|  | Tonsillitis | 1 (0.3%) | 1 (0.1%) |
|  | Traumatic fracture | 1 (0.3%) | 1 (0.1%) |
|  | Tremor | 1 (0.3%) | 1 (0.1%) |
|  | Urinary tract obstruction | 1 (0.3%) | 1 (0.1%) |
|  | Vestibular disorder | 1 (0.3%) | 1 (0.1%) |
|  | Viral infection | 1 (0.3%) | 2 (0.3%) |
| adverse_event_type/seizure | Epilepsy | 1 (0.3%) | 1 (0.1%) |
| adverse_event_type/tract_infection | Respiratory tract infection | 21 (5.4%) | 25 (3.4%) |
|  | Pneumonia | 4 (1%) | 4 (0.5%) |
|  | Nasopharyngitis | 3 (0.8%) | 3 (0.4%) |
|  | COVID-19 | 2 (0.5%) | 2 (0.3%) |
|  | Cough | 2 (0.5%) | 2 (0.3%) |
|  | Bronchitis | 1 (0.3%) | 1 (0.1%) |
|  | Influenza like illness | 1 (0.3%) | 1 (0.1%) |
|  | Pyrexia | 1 (0.3%) | 1 (0.1%) |
|  | Respiratory tract infection viral | 1 (0.3%) | 1 (0.1%) |
|  | Rhinolaryngitis | 1 (0.3%) | 1 (0.1%) |
|  | Upper respiratory tract infection | 1 (0.3%) | 1 (0.1%) |
|  | Viral infection | 1 (0.3%) | 1 (0.1%) |
|  | Viral upper respiratory tract infection | 1 (0.3%) | 1 (0.1%) |
|  |  |  |  |
